# Analysis of the Structural Validity of the Reduced Version of Metacognitive Awareness of Reading Strategies Inventory

**DOI:** 10.3389/fpsyg.2022.894327

**Published:** 2022-06-14

**Authors:** Daniel Ondé, Virginia Jiménez, Jesús M. Alvarado, Marta Gràcia

**Affiliations:** ^1^Department of Psychobiology and Behavioral Sciences Methods, Faculty of Psychology, Complutense University of Madrid, Madrid, Spain; ^2^Department of Experimental Psychology, Cognitive Psychology and Speech and Language Therapy, Faculty of Social Work, Complutense University of Madrid, Madrid, Spain; ^3^Department of Cognition, Development and Psychology of Development, Faculty of Psychology, University of Barcelona, Barcelona, Spain

**Keywords:** metacognition, reading comprehension, metacognitive awareness, strategies, bifactor S-1

## Abstract

The application of metacognitive strategies is considered a basic skill of the student at any educational level. In the present study, we evaluate the reduced version of the Metacognitive Awareness of Reading Strategies Inventory (MARSI-R) in Spanish, a self-report instrument designed to measure the metacognitive awareness of students and their perception of the strategies that they use while they are reading school materials. MARSI-R is formed by three subscales: (a) global reading strategies (GRS), (b) problem-solving strategies, and (c) strategies to support reading. We conducted a confirmatory factor analysis (CFA) in a Spanish student sample (*N* = 570) and the results shown relative inadequate fit for the proposed theoretical three-factor model. More important, the three subscales presented a high level of inter-correlation, which raises the need to assess to what extent the construct should be considered as unidimensional. We conducted two additional CFA models: a unidimensional model and a bifactor S-1 model, and the results indicated the presence of a strong general factor related to the GRS subscale. These results have important implications, since they imply that it is more appropriate to use the total score of the instrument derived of the S-1 model instead of the scores derived from each subscale. The bifactor S-1 model has allowed us to develop a closer approximation between the psychometric model and the theoretical model.

## Introduction

Metacognition can be defined as the knowledge and control of one’s cognitive activity. This metacognitive competence is the basis of “learning to learn” and understanding (Coll et al., 2012). The development of metacognitive strategies in the student will develop their self-regulated learning. Flavell (1976), a pioneer of the term, defined metacognition as self-knowledge concerning one’s cognitive processes and products or everything related to them. Later, he added motivational and affective components to the definition (Flavell, 1979; Paris and Winograd, 1990).

The concept of metacognition includes three types of knowledge: (a) declarative (referring to the strategies used to learn), (b) procedural (steps to use the chosen strategies), and (c) conditional (when, where and why the chosen strategies are used instead of others), and its self-regulation through the processes of planning, selection of strategies, and evaluation of learning or monitoring. These types of metacognitive knowledge and strategies are studied in many learning fields. In this work, we focus on the field of reading (see Jiménez et al., 2009; Soto et al., 2019; Deliany and Cahyono, 2020; Yong and Abdul, 2020).

One of the basic tools for learning knowledge is reading (more specifically, reading comprehension; for example, Solé et al., 2005; Mateos, 2009). To become a competent reader, two types of skills are needed: cognitive and. The latter are the ones that allow awareness and control of the comprehension process. Good readers are characterized by the possession of a series of monitoring and revising strategies for approaching a text, by a certain degree of awareness of their reading methods and the demands of the task, and by making use of the context (i.e., reading metacomprehension).

Among the different instruments developed for the reading metacomprehension measurement (see Soto et al., 2018), the MARSI (Mokhtari and Reichard, 2002) stands out due to its ease of application, especially in its short version: MARSI-R (Mokhtari et al., 2018), which makes it ideal for quick screening. Original and shortened versions of the instrument are proposed to evaluate the same three strategies domains: global reading strategies (GRS, focused on a global analysis of the text), problem-solving strategies (PSS, used in situations when parts of the text seem difficult to read), and support reading strategies (SRS, support strategies such as using reference materials or taking notes). These strategies are activated when the text presents a certain degree of understanding difficulty for the reader. In Supplementary Material, we show the MARSI-R items (original and Spanish versions).

The MARSI-R has shown better psychometric properties than the original version when selecting the most discriminating items of each dimension (see Deliany and Cahyono, 2020; Yong and Abdul, 2020; Rahimi and Abolfazl, 2021). In addition, a good fit to the proposed three-dimensional structure and evidence of validity regarding other relevant variables, specifically the correlation between the total score and the self-reported reading level (“READER”), have been observed. As Reise (2012, p. 14) points out, “the presence of multidimensionality, *per se*, does not necessarily muddy the interpretability of a unit-weighted composite score, nor does it automatically demand the creation of subscales. Thus, researchers must make a distinction between the degree of unidimensionality in the data, and the degree to which total scores reflect a single common variable.”

The MARSI-R supports the possibility of obtaining a global score and a score for each reading strategies (GRS, PSS, and SRS). For the use of the total score, however, it is necessary to show that its structure is, at least, essentially unidimensional (see Trizano-Hermosilla et al., 2021). If there is not enough common variance for the 15 items of the instrument, the use of the total score should be discarded and instead used as a multidimensional instrument. In the study by Mokhtari et al. (2018) the correlations between the three proposed factors were highly correlated (i.e., factor correlations between 0.618 and 0.840). This evidence raises the need to assess whether the measure is unidimensional or essentially unidimensional, an issue that has not been addressed to date.

In the present study, we evaluate the factorial structure of the MARSI-R in the Spanish population. Using CFA, we have compared the original three-factor structural model with a unidimensional model and a bifactor S-1 model to assess the degree of unidimensionality of the test. The unidimensional model is a common CFA single-factor model that fix all correlations between item error terms to zero. The three-factor model is a common CFA factor-correlated structure that fix all cross-loadings and all correlations between item error terms to zero (see Brown, 2015 for technical details). In Supplementary Material we show conceptual path diagrams of both models.

**FIGURE 1:**
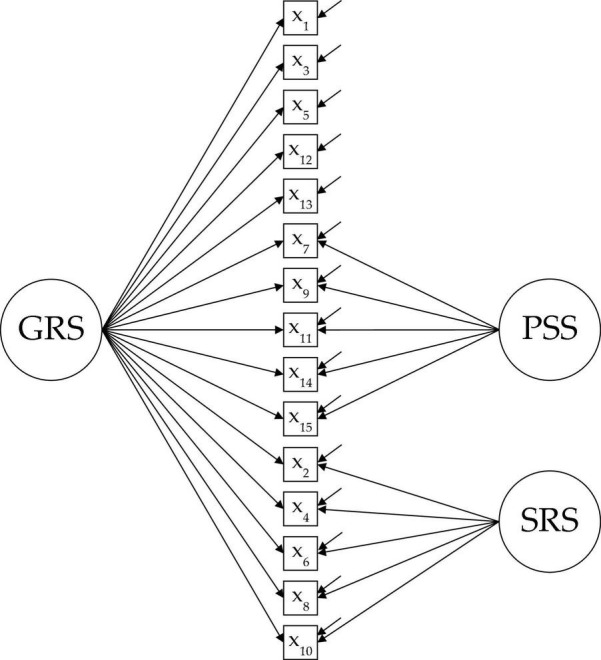
Path-diagram of bifactor S-1 model (global reading strategies as reference general factor). GRS, global reading strategies; PSS, problem solving strategies; SRS, support reading strategies.

The bifactor S-1 model specifies one of the item cluster of contents, domain, or group factor as a general factor (i.e., variance common to all items) and analyzes the rest of the factors controlled for the variance due to the general factor. This model allows simultaneously assessing the importance of the general factor (i.e., unidimensionality) versus the different group factors (i.e., multidimensionality). This information only is partially assessed with each of the previous models: variance common to all items using the unidimensional model (i.e., strict unidimensionality), or specific variance of each group factor using the three-factor model (i.e., multidimensionality). Given the very nature of the instrument, we consider that the psychometric model that can best capture the structure of the scores is a bifactor S-1 model in which the GRS factor is used as a general reference factor, keeping the PSS and SRS factors as group factors (see Figure 1). The bifactor S-1 model has shown good performance in other evaluation contexts, for example, attention-deficit/hyperactivity disorder (ADHD) and oppositional defiant disorder (ODD) symptoms (Burns et al., 2020), intelligence (Eid et al., 2018), and emotional intelligence (Ondé et al., 2021).

## Methods

### Participants

The sample consisted of 570 students (41.9% male, 54.0% female, and 4.1% other) of Compulsory Secondary Education from various educational centers of Barcelona and Madrid (Spain). The educational centers were selected by convenience, so we applied a non-probabilistic sampling method. A total of 27.1% of the participants were first grade, 32.1% were second grade and 40.2 were third grade students (mean age = 14.3 years, SD = 1.0, range between 13 and 17 years old). As Mokhtari et al. (2018, p. 222), there exists within any grade (or classroom) a range of readers and a range of reading ability levels. In this sense, the sample of this study is composed of typical readers within the grade in which they are, with general academic abilities (i.e., general student population).

### Measures

MARSI-R (Mokhtari et al., 2018; see Supplementary Material) is a self-report instrument designed to assess the awareness of reading strategies while reading school-related texts of students ranging from 11 to 18 years old. This instrument maintains the same structure of three factors proposed in the original version (MARSI; Mokhtari and Reichard, 2002), with five items in each factor (GRS, PSS, and SRS) with five ordered responses categories. The test also includes an item about self-perceived reading level (henceforth READER variable) that records how excellent (or not) the students view themselves concerning reading, with four ordered responses categories (1. A poor reader, 2. An average reader, 3. A good reader, and 4. An excellent reader). Mokhtari et al. (2018) state that MARSI-R avoids a certain degree of overlap between items identified in the original version. In addition, the length of the reduced version is more affordable for the target population, being able to avoid bias in responses, dropouts, etc.

### Procedure

We contact educational centers and inform the directors and the teachers about the study and its objectives. The director of each center reported to the parents of the students about the study. Then, the parents were asked to give their informed consent to conduct the data collection with the students. Afterward, teachers administered the MARSI-R in the classroom during a typical daily class session (the tutoring hour, a common type of session within the Spanish Educational System).

### Statistical Analyses

First, we conducted a series of preliminary analyses, which include the common descriptive statistics of the items [mean, standard deviation (SD), skewness, and kurtosis]. Second, we conducted three CFA to fit the following models: unidimensional (UNI), the original three correlated factors (3CORR), and bifactor S-1 with GRS as general reference factor [S-1(GRS)]. We used the polychoric correlation matrix as the input matrix for CFA, appropriate when analyzing ordinal variables (i.e., items; see Yang-Wallentin et al., 2010; Brown, 2015). We used the lavaan package for the R program (Rosseel, 2012), and diagonal weighted least squares (DWLS) as the estimation method (Asún et al., 2016).

To examine the CFA-model fit we used the χ^2^ difference between models test (Δχ^2^), the root mean square error of approximation (RMSEA), the standardized root mean square residual (SRMR), the comparative fit index (CFI), and the Tucker–Lewis index (TLI). We used the following criteria for evaluating model fit (see, for example, Brown, 2015): Δχ^2^ *p*-value, RMSEA < 0.08, the test of close fit of RMSEA (*p*-value), SRMR close to 0.08 or below, CFI and TLI > 0.95.

To evaluate the degree of unidimensionality and reliability (i.e., internal consistency) of the MARSI-R measure we used the explained common variance (ECV) index and the hierarchical omega coefficient (ω*_*H*_*). ECV is the common variance explained by a factor divided by the total common variance. ω*_*H*_* reflects only variance attributable to a single factor and is computed by dividing the squared sum of the factor loadings on the general factor by the estimated variance of total scores. Both are model-based indices and must be used when the model reflects a hierarchical structure, as the S-1 model. To consider a measure as essentially unidimensional, it has been recommended to obtain ECV values higher than 0.60 −0.70 and ω*_*H*_* > 0.70 (for technical details, see Reise et al., 2013; Rodriguez et al., 2016a,b). For UNI and 3CORR models, we used coefficients omega (ω) and omega subscale (ω*_*S*_*), model-based indices that estimate the proportion of variance attributable to all sources of common variance (McDonald, 2013). We used the BifactorIndicesCalculator R package (an R version of the Excel-based Bifactor Indices Calculator; Dueber, 2017) to calculate the ECV, ω*_*H*_*, ω, and ω*_*S*_* values.

Finally, we estimate the predictive capacity that MARSI-R has on the READER variable using CFA.

## Results

Item-mean ranges between 2.8 and 4.2. The lowest SD was 1.2, and the highest was 1.4. Most asymmetry indices range between ± 1 (except item 8: asymmetry = −1.6). All kurtosis indices range between 0 and −1.5.

The fit of the 3CORR model is statistically better than that of the UNI model (significant χ^2^ difference test, see Table 1) with very few differences in the rest of the benchmarks. The correlations between the three factors of the 3CORR model are very high, ranging between 0.804 (PSS–SRS) and 0.864 (GRS–PSS). In the study by Mokhtari et al. (2018), the correlations ranged between 0.618 and 0.840. The model that best fits is the S-1(GRS) model, with a statistically better fit than the 3CORR model (χ^2^ difference test = 67.8; *p* < 0.01).

**TABLE 1:** Goodness-of-fit indices for the UNI, 3CORR, and S-1(GRS) models.

Model	χ^2^ (df)	CFI	TLI	RMSEA (90% CI)	Test of close fit (RMSEA)	SRMR	Δχ^2^ (Δdf)
UNI	316.5 (90)**	0.951	0.943	0.067 (0.059–0.075)	<0.001	0.067	–
3CORR	284.2 (87)**	0.958	0.949	0.063 (0.055–0.071)	0.004	0.064	33.6 (3)**
S-1(GRS)	223.0 (80)**	0.969	0.960	0.056 (0.047–0.065)	0.122	0.056	67.8 (7)**

Δχ^2^, χ^2^ difference between models; Δdf, difference between degrees of freedom; p-value codes: **p < 0.01.

The value of ω for the UNI model is ω_*UNI*_ = 0.816 and for the three factors of the 3CORR model were ω_*GRS*_ = 0.609, ω_*PSS*_ = 0.667, and ω_*SRS*_ = 0.647. For the S-1(GRS) model, the ECV value for the general factor GRS is 0.728 and the value of ω*_*H*_* is 0.779. The ECV for PSS and SRS was 0.359, and 0.370, respectively. The ω*_*H*_* for these group factors was 0.177 and 0.127, respectively. Following the recommendations of Reise et al. (2013) and Rodriguez et al. (2016a,b) on the ECV and ω*_*H*_*, these results indicate that the measurements obtained by the MARSI-R can be considered essentially unidimensional.

**FIGURE 2:**
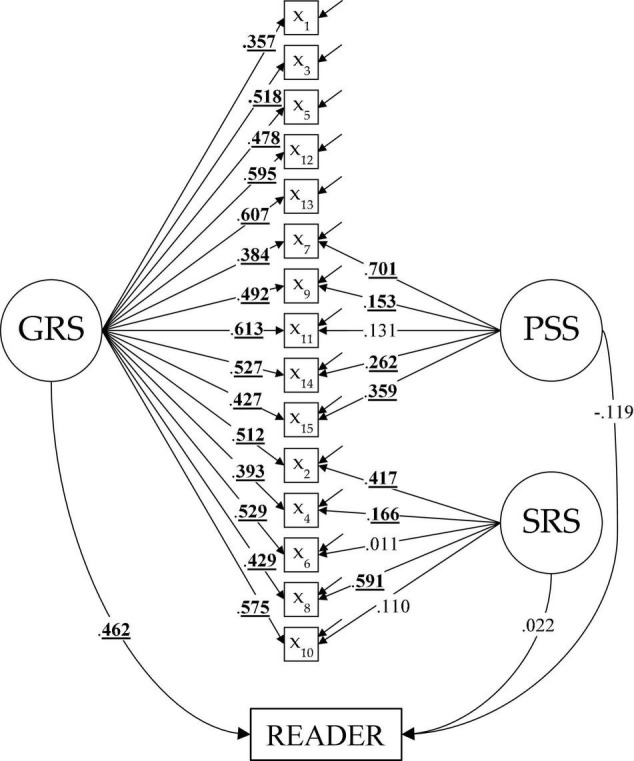
Confirmatory factor analysis parameter estimates of the S-1(GRS) model by regressing READER variable onto latent factors (GRS, PSS, and SRS).

Finally, we extend the CFA from the S-1(GRS) model by including the READER variable in the model, which has allowed us to evaluate the predictive capacity of the instrument. Figure 2 shows the result of this analysis, including the factor loadings of the S-1(GRS) model and the regression coefficients obtained by regressing READER variable onto latent factors (the values underlined and in bold are those that have been statistically significant (*p* < 0.05); in Supplementary Material we show CFA parameter estimates for UNI and 3CORR models). It is observed that the general reference factor (GRS) predicts reader scores (β_*GRS*_ = 0.462; *p* < 0.05), while the group factors do not have a statistically significant predictive value (β_*PSS*_ = −0.119 and β_*SRS*_ = 0.022). These results are consistent with those found by Mokhtari et al. (2018), who found a statistically significant correlation of 0.330 between the total MARSI-R score and the READER variable. However, our results differ from those found by Mokhtari et al. (2018), who showed statistically significant correlations between PSS and READER (0.346) and SRS and READER (0.163). Given that in the three-factor model the factor inter-correlations are very high, it seems reasonable to think that the predictive capacity of the subscales reflects the relationship between the common variance to all the items (i.e., a general factor). Once we control the variance common to all the items [S-1(GRS) structure], the group factors show no significant predictive capacity over the READER variable.

## Discussion

The MARSI-R has shown an adequate approximation to the S-1 model, and this result has important theoretical and applied implications. Regarding the theoretical implications, the original instrument and its shortened version were designed to assess metacomprehension by tracking three content domains, one referring to general strategies and two to specific ones. Consequently, the theoretical model ideally corresponds to an S-1 model that allows capturing the hierarchical relationship between general (GRS) and specific (PSS and SRS) competencies. Regarding the applied implications, the factorial structure indicates how the scores of an instrument can and should be used, whether it can be used as a single scale or as a multidimensional instrument (Trizano-Hermosilla et al., 2021). Therefore, only if the instrument is unidimensional or essentially unidimensional the use of the total score as a measure of a reflective construct is legitimate (Rodriguez et al., 2016b).

Metacomprehension is the basis for educational self-regulation, learning and academic performance (Donker et al., 2014; Bittner et al., 2021). MARSI-R is an instrument that can be useful in assessing metacomprehension. However, some issues should be considered. First, our results show that the original structure proposed of three domains or clusters of reading strategies do not reach values that clearly indicate goodness of fit. Second, ω values are below 0.65, so the scores derived from each subscale do not reach adequate levels of internal consistency. Third, and more important, the high values of factor inter-correlations suggest the possibility of considering the measure of the construct as unidimensional. This question has not been addressed to date in previous studies. We evaluated the strict and essential unidimensionality using unidimensional and S-1 CFA models, respectively. The S-1(GRS) model shows better performance compared to the unidimensional model. On the one hand, this model is the only one that obtains a good fit. On the other hand, the general factor GRS is well enough determined to consider the measure as essentially unidimensional (ECV and ω*_*H*_* higher than 0.70).

### Limitations and Future Research

First, the global GRS score of the MARSI-R has shown the ability to predict responses to the READER variable, a self-reported reading level. It would be convenient to conduct additional studies to analyze the relationship of the MARSI-R score with other criterion variables to improve the level of evidence related to predictive validity. Second, although the sample size is acceptably large, it would not be appropriate to overgeneralize our results. New studies with new samples (and other educational centers) are needed to verify our findings. Third, when factor inter-correlations are high, as in our case, it is legitimate to conceive the measure as essentially unidimensional (Reise, 2012), which also legitimizes the use of the general factor score as a global score for prediction or psychological diagnosis. The correlations between factors observed by Mokhtari et al. (2018) are also high. This situation leads us to recommend that future studies consider evaluating both the degree of unidimensionality (considering the GRS factor as a general reference factor) and the original three-factor structure of the MARSI-R in other cultures. As a methodological note, just as it is recommended not to induce the reliability of a scale based on the results of previous studies, neither should its factorial structure be induced, and it should be contrasted in each new application to increase (if possible) the level of evidence related to the internal structure. Fourth, this brief paper focuses on methodological aspects related to the structural or factorial validity of the MARSI-R and the most appropriate use that can be made of the scores derived from the instrument. Therefore, its scope is limited since it leaves out relevant aspects of the evaluation of metacomprehension in reading for Spanish students. It would be interesting to conduct other studies that delve into the degree of adaptation of the short version of the MARSI in the Spanish population, simultaneously considering other evaluation instruments (see, for example, Soto et al., 2018).

## Conclusion

This work is useful to characterize the MARSI-R measure within the Spanish context. In the present study, we evaluate the factorial structure of the MARSI-R in the Spanish population, comparing the original three-factor structural model with a unidimensional model and a bifactor S-1 model. S-1(GRS) is the model that best reflects the real characteristics of the participants’ responses since it allows unidimensionality to be assumed in the presence of a certain degree of multidimensionality (i.e., thanks to the S-1 model we can obtain a more refined and better determined global latent score by controlling for the variance due to the group factors). In this sense, and regardless of whether the three-factor model fits or not, the conceptualization of the S-1(GRS) model has proven to be superior since it allows a better understanding of the three-factor correlations. The results presented in this study lead us to recommend the use of a single global score (i.e., factor scores of the GRS as a general reference factor), avoiding the use of the three subscale scores.

## Data Availability Statement

The raw data supporting the conclusions of this article will be made available by the authors, without undue reservation.

## Ethics Statement

The studies involving human participants were reviewed and approved by the Institutional Review Board of the University of Barcelona (protocol code IRB00003099 and 21 December 2020) and the Deontological Committee of Faculty of Psychology, Complutense University of Madrid (2020/21-007, 29 October 2020). Written informed consent to participate in this study was provided by the participants’ legal guardian/next of kin.

## Author Contributions

VJ, JA, DO, and MG: development of the idea of research and theoretical framework and discussion of the results in light of current theoretical background. DO and VJ: construction of the methodological framework. DO and JA: data analysis and interpretation of data. MG and VJ: revision. All authors contributed to the article and approved the submitted version.

## Conflict of Interest

The authors declare that the research was conducted in the absence of any commercial or financial relationships that could be construed as a potential conflict of interest.

## Publisher’s Note

All claims expressed in this article are solely those of the authors and do not necessarily represent those of their affiliated organizations, or those of the publisher, the editors and the reviewers. Any product that may be evaluated in this article, or claim that may be made by its manufacturer, is not guaranteed or endorsed by the publisher.
