# Supplementary material for: Analysis of the Structural Validity of the Reduced Version of Metacognitive Awareness of Reading Strategies Inventory
Source: Front Psychol. 2022 Jun 14;13:894327. doi: 10.3389/fpsyg.2022.894327 (PMC9237459; doi:10.3389/fpsyg.2022.894327)
Supplement: Supplementary file 1 [file Presentation_1.pdf]

Metacognitive Awareness of Reading Strategies Inventory-Revised (MARSI-R)

English and Spanish versions

[LECTOR] I consider myself (Check one):

(Spanish translation in parentheses)

1. An excellent reader (*Un/a lector/a excelente*).
2. A good reader (*Un/a buen/a lector/a*).
3. An average reader (*Un/a lector/a medio*).
4. A poor reader (*Un/a pobre lector/a*).

Read each statement to indicate whether you are aware of and/or use these strategies when you read. Use the following scale to show your strategy awareness and/or use (*Lee cada oración para indicar si eres consciente y/o utilizas esas estrategias cuando lees. Utiliza la escala siguiente para señalar la conciencia y/o uso que tienes sobre la estrategia de la lista*):

1. I have never heard of this strategy before (*Nunca había oído hablar de esta estrategia*).
2. I have heard of this strategy, but I don't know what it means (*He oído hablar de esta estrategia, pero no sé lo que significa*).
3. I have heard of this strategy, and I think I know what it means (*He oído hablar de esta estrategia y creo que sé lo que significa*).
4. I know this strategy, and I can explain how and when to use it (*Conozco esta estrategia y puedo explicar cómo y cuándo utilizarla*).
5. I know this strategy quite well, and I often use it when I read (*Conozco esta estrategia bastante bien y la utilizo a menudo cuando leo*).

[We indicate the content domain of each item (i.e., factor) in parentheses. GRS – Global reading strategies; PSS – Problem solving strategies; SRS – Support reading strategies]

1. (GRS) Having a purpose in mind when I read (*Tener un propósito en mente cuando se lee*).
2. (SRS) Taking notes while reading (*Tomar notas (apuntes) mientras se lee*).
3. (GRS) Previewing the text to see what it is about before reading it (*Echar un vistazo al texto para saber de qué trata antes de leerlo*).
4. (SRS) Reading aloud to help me understand what I'm reading (*Leer en voz alta como ayuda para entender la lectura*).
5. (GRS) Checking to see if the content of the text fits my purpose for reading (*Comprobar que el contenido del texto se ajusta al propósito de lectura*).
6. (SRS) Discussing what I read with others to check my understanding (*Discutir lo leído con otras personas para comprobar la comprensión que se tiene de lo leído*).
7. (PSS) Getting back on track when getting sidetracked or distracted (*Darse cuenta de que se ha perdido la comprensión del texto por una distracción y volver a leer desde donde se perdió la comprensión*).
8. (SRS) Underlining or circling important information in the text (*Subrayar o rodear con un círculo la información importante del texto*).
9. (PSS) Adjusting my reading pace or speed based on what I'm reading (*Ajustar el ritmo de lectura o la velocidad lectora dependiendo de lo que se lee*).
10. (SRS) Using reference materials such as dictionaries to support my reading (*Utilizar materiales de referencia como diccionarios para apoyar la lectura*).
11. (PSS) Stopping from time to time to think about what I'm reading (*Parar la lectura de vez en cuando para pensar sobre lo que se está leyendo*).
12. (GRS) Using typographical aids like bold face and italics to pick out key information (*Usar ayudas tipográficas como negrita o cursiva para marcar la información clave*).
13. (GRS) Critically analyzing and evaluating the information read (*Analizar de forma crítica y evaluar la información leída*).
14. (PSS) Re-reading to make sure I understand what I'm reading (*Releer para asegurar la comprensión de la lectura*).
15. (PSS) Guessing the meaning of unknown words or phrases (*Adivinar el significado de palabras o frases desconocidas*).

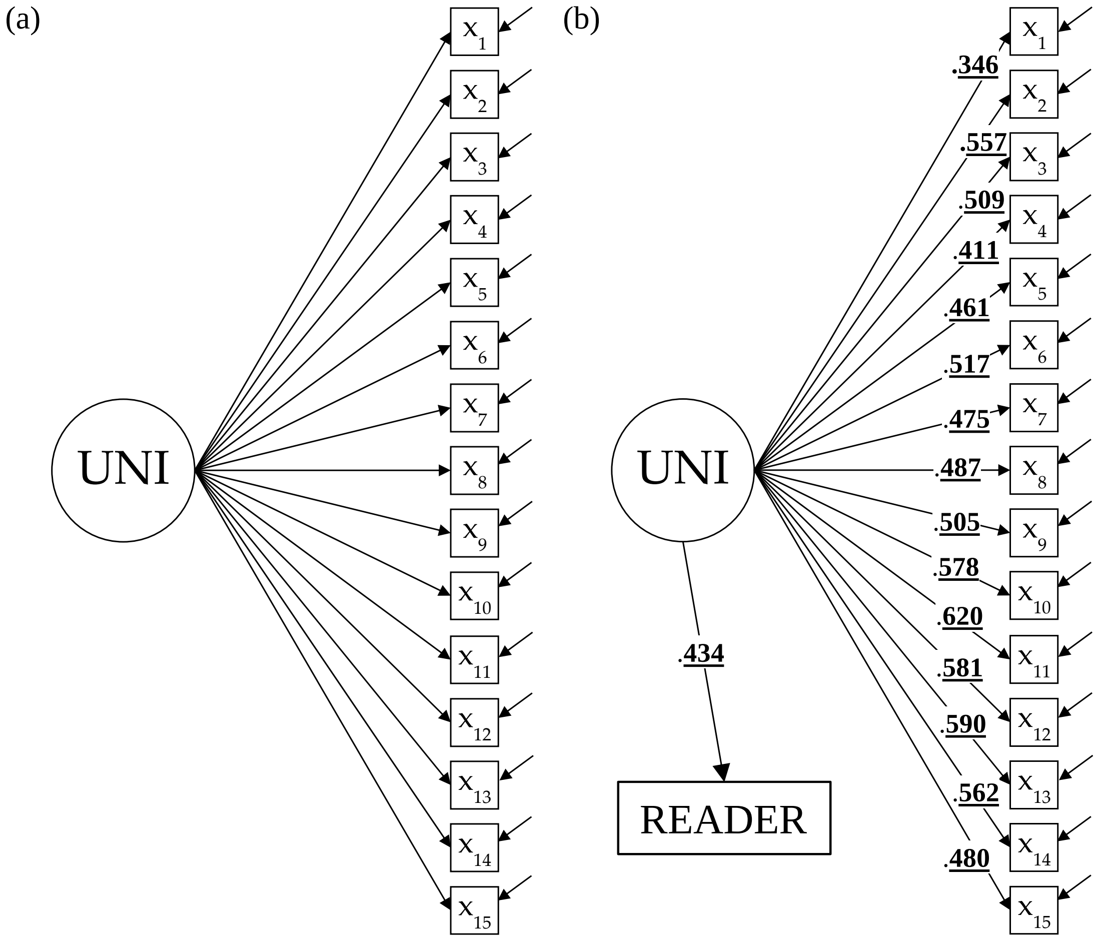

Figure A.1. Path-diagram of (a) UNI model (a single-factor CFA model) and (b) CFA parameter estimates by regressing READER variable onto latent factor. The values underlined and in bold are those that have been statistically significant ($p < .05$).

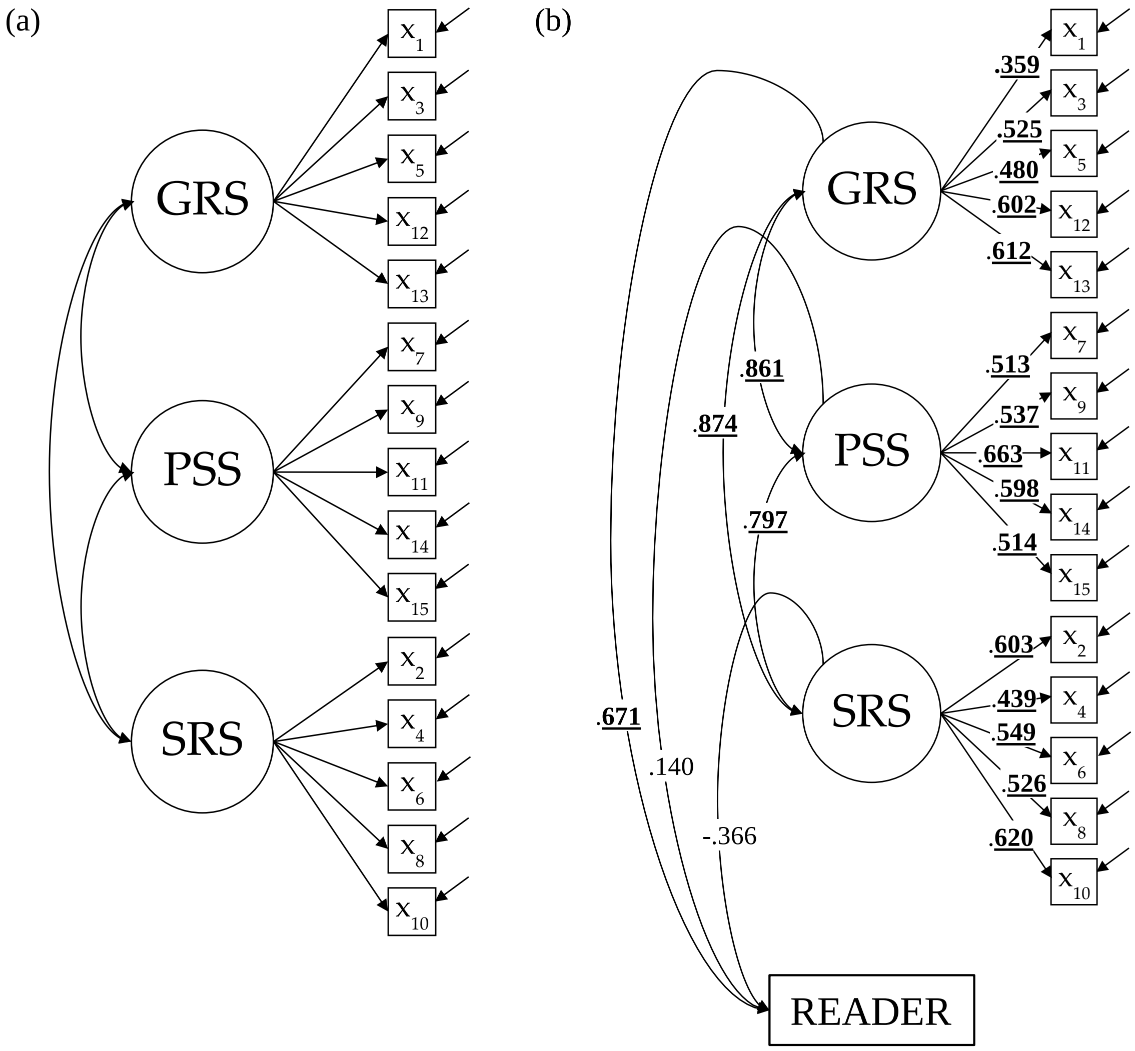

Figure A.2. Path-diagram of (a) original three-factor model proposed by Mokhtari et al. (2018) and (b) CFA parameter estimates by regressing READER variable onto latent factors (GRS, PSS and SRS). GRS – Global reading strategies; PSS – Problem solving strategies; SRS – Support reading strategies. The values underlined and in bold are those that have been statistically significant ($p < .05$).
